# Artificial Intelligence–Assisted Surgical Scene Recognition: A Comparative Study Among Health Care Professionals

**DOI:** 10.1097/SLA.0000000000006577

**Published:** 2024-10-30

**Authors:** Simon C. Williams, Jinfan Zhou, William R. Muirhead, Danyal Z. Khan, Chan Hee Koh, Razna Ahmed, Jonathan P. Funnell, John G. Hanrahan, Alshaymaa Mortada Ali, Shankhaneel Ghosh, Tarik Saridoğan, Alexandra Valetopoulou, Patrick Grover, Danail Stoyanov, Mary Murphy, Evangelos B. Mazomenos, Hani J. Marcus

**Affiliations:** * UCL Hawkes Institute, University College London, London, UK; † Victor Horsley Department of Neurosurgery, National Hospital for Neurology and Neurosurgery, London, UK; ‡ Department of Robotics, University of Michigan, Ann Arbor, MI; § Ain Shams University, Cairo, Egypt; ‖ Department of General Medicine, Institute of Medical Sciences and SUM Hospital, Bhubaneswar, India; ¶ Department of Emergency Medicine, Bursa City Hospital, Bursa, Turkey; # Department of Neurosurgery, Imperial College Healthcare NHS Trust, London, UK

**Keywords:** aneurysm, artificial intelligence, computer vision, machine learning, neurosurgery, vascular neurosurgery

## Abstract

**Objective:**

To compare the ability of a deep-learning platform (the MACSSwin-T model) with health care professionals in detecting cerebral aneurysms from operative videos. Secondly, we aimed to compare health care professionals' ability to detect cerebral aneurysms with and without artificial intelligence (AI) assistance.

**Background:**

Modern microscopic surgery enables the capture of operative video data on an unforeseen scale. Advances in computer vision, a branch of AI, have enabled automated analysis of operative video. These advances are likely to benefit clinicians, health care systems, and patients alike, yet such benefits are yet to be realized.

**Methods:**

In a cross-sectional comparative study, neurosurgeons, anesthetists, and operating room nurses, all at varying stages of training and experience, reviewed still frames of aneurysm clipping operations and labeled frames as “aneurysm not in frame” or “aneurysm in frame.” Frames then underwent analysis by the AI platform. A second round of data collection was performed, whereby the neurosurgical team had AI assistance. The accuracy of aneurysm detection was calculated for human-only, AI-only, and AI-assisted human groups.

**Results:**

A total of 5154 individual frame reviews were collated from 338 health care professionals. Health care professionals correctly labeled 70% of frames without AI assistance, compared with 78% with AI assistance (odds ratio: 1.77, *P* < 0.001). Neurosurgical Attendings showed the greatest improvement, from 77% to 92% correct predictions with AI assistance (odds ratio: 4.24, *P* = 0.003).

**Conclusions:**

AI-assisted human performance surpassed both human and AI alone. Notably, across health care professionals surveyed, frame accuracy improved across all subspecialties and experience levels, particularly among the most experienced health care professionals. These results challenge the prevailing notion that AI primarily benefits junior clinicians, highlighting its crucial role throughout the surgical hierarchy as an essential component of modern surgical practice.

Artificial intelligence (AI) offers novel solutions to surgical problems.^1,2^ Computer vision (CV), a domain of AI, enables images and videos to be analyzed and contextualized by computer technology.^1^ Advances in computer vision analysis of radiographic and diagnostic imaging modalities have been significant in the past two decades, yet these benefits are yet to be translated to the operating room (OR).^3^ Increasingly, however, intraoperative video is being viewed as a mass of untapped data with huge potential. Indeed, recent advancements in intraoperative CV include phase recognition,^4,5^ navigation,^6^ and instrument segmentation.^7^ In this study, we aimed to demonstrate the benefit of surgical computer vision, using microsurgical clipping of cerebral aneurysms as an exemplar.

Surgical clipping of cerebral aneurysms, particularly ruptured aneurysms, is a high-risk procedure, with 30% of operations experiencing complications and 36% of poor outcomes attributable to intraoperative issues.^8,9^ The most feared intraoperative complication is aneurysm rupture, occurring in 17% of cases.^8^ Crucially, the greatest risk of aneurysm rupture occurs when the aneurysm is in the surgical field of view.^8^ Intraoperative identification of aneurysms can be challenging owing to narrow surgical corridors, anatomic variation, surrounding vasculature, and dense arachnoid adhesions. Indeed, interpreting three-dimensional anatomy in real-time and identification of small cerebral aneurysms feature among the top technical challenges faced by neurovascular surgeons.^10^ Recognition of cerebral aneurysms is not only relevant to the operating surgeon but also to the wider theater team. Effective teamwork among the surgical team is essential to reducing the risk of intraoperative complications. Intraoperative cohesion and understanding improve efficiency, reduce stress burden, and facilitate rapid solving of intraoperative problems in what are frequently dynamic and high-stress scenarios.^11,12^ Indeed, communication breakdowns and misunderstandings between surgical team members have been shown to contribute to adverse events.^11,13^ The notion of ‘shared mental models’, in which a team shares a collective understanding of a situation, helps elucidate why group understanding is paramount to intraoperative safety.^14,15^ Essential to this shared experience is scene recognition. Put simply, it is important that all personnel in theater environments understand when the highest risk phase of an operation is occurring, such as the aneurysm clipping phase. Recognition enables preparedness and heightened alertness among the surgical team and safeguards against unnecessary distraction, akin to the “sterile cockpit” protocol adopted in the aviation industry.^16,17^ Innovations to increase scene recognition and awareness, therefore, stand to benefit patient safety.

Previously, our group described MACSSwin-T, a deep-learning platform able to detect or exclude the presence of cerebral aneurysms inoperative video, utilising a Shifted-Windows Transformer architecture.^18^ MACSSwin-T achieved an accuracy of 80.8% (precision 51.3% and recall 63.8%) and an average F1 score of 56.8% in multiple cross-fold validation.^18^ An optimized model was produced, and an initial expert validation assessment demonstrated noninferiority when compared with a cohort of 10 attending (consultant grade) neurosurgeons.^18^

Surgical technologies benefit from a stepwise evaluation, such as described by the Idea, Development, Exploration, Assessment, Long Term (IDEAL) Framework.^19^ Before first-in-human studies, a range of factors pertaining to the safety and efficacy of an innovation should be explored. This comparative, ex vivo (IDEAL stage 0) study builds on our previous work by comparing the efficacy of the MACSSwin-T platform against neurosurgical health care professionals in detecting cerebral aneurysms from microsurgical aneurysm clipping operations. This study aimed to validate our intervention by comparing the MACSSwin-T platform with neurosurgical health care professionals in identifying cerebral aneurysms in microsurgical clipping operations. Secondly, we aimed to compare neurosurgical health care professional’s ability to detect cerebral aneurysms with and without AI assistance. In doing so, we aim to demonstrate the benefits of computer vision in surgical contexts.

## METHODS

### Overview of Methods

An online survey was created and distributed to neurosurgical health care professionals (neurosurgeons, anesthetists, and OR nurses) worldwide, all at varying stages of training and experience. Participants reviewed 15 still frames (7 containing an aneurysm and 8 without an aneurysm) derived from 4 aneurysm clipping videos and determined whether each frame contained an aneurysm. The frames were analyzed by the MACSSwin-T platform, which predicted whether the frames did or did not contain an aneurysm. Human and AI performance results were compared. In a second version of the survey, the neurosurgical health care professionals received AI assistance, and initial human performance was compared with AI-assisted performance. The survey methodology adhered to Good Survey Practice guidelines^20^ and has been reported in keeping with CROSS guidelines.^21^ This comparative validation study represents an IDEAL stage 0 study.^19^ At the time of publication, TRIPOD-AI reporting guidelines were not published—in the absence of these, this study has adhered to TRIPOD guidelines where applicable.^22^ All patients provided written informed consent for the research video recordings, adhering to General Medical Council guidelines; videos and images were anonymized, and formal governance approval was obtained (Registration Reference 85-202021-SE).

### Model Development: Microsurgical Aneurysm Clipping Surgery Data Set and the MACSSwin-T Platform

Zhou et al^18^ created a data set of 16 aneurysm clipping operative videos with expert-labeled annotations, which was used to train a deep-learning architecture for detecting cerebral aneurysms. Frames were extracted from 16 operative videos at a rate of 5 frames per second, resulting in a data set of 356,165 images (the Training Data Set—publicly available at available online: https://doi.org/10.5522/04/23533731).^23^ All frames were labeled by experts as containing (n = 71,113 frames) or not containing an aneurysm (n = 285,052), and labeled frames were used to train a deep-learning platform (MACSSwin-T, details of which can be found in the original publication^18^) in a supervised learning phase. The MACSSwin-T platform utilizes a shifted-windows (Swin-T) transformer architecture to classify frames. MACSSwin-T is based on hierarchical, multiscale self-attention which allows the generation of localized features from multiple frames. These were then aggregated enabling the platform to detect and distinguish the aneurysm from similar-looking adjacent vasculature. The MACSSwin-T platform’s function was exclusively to identify cerebral aneurysms from operative video; the platform had no bearing on patient selection for treatment. The MACSSwin-T model underwent 4-fold cross-validation in a 12:4 training:test split, achieving an accuracy of 80.8% (precision 51.3% and recall 63.8%). In an initial expert validation assessment, the platform demonstrated noninferiority when compared with a cohort of 10 attending neurosurgeons.^18^

### Survey Development

Frames of operative aneurysm clippings used in the online survey were taken from a secure database of operative video recordings (1280×1080 pixels) of 4 patients obtained at a single tertiary academic center in the UK between 2020 and 2021. Videos were derived from elective and emergency cases. No criteria were applied regarding the location or morphology of the aneurysm, or the type of surgical clip applied. Operative videos were recorded directly to a ZEISS Kinevo 900 operating microscope (Carl Zeiss Co.), or a ZEISS OPMI Pentero 800 operating microscope (Carl Zeiss Co.).

**FIGURE 1:**
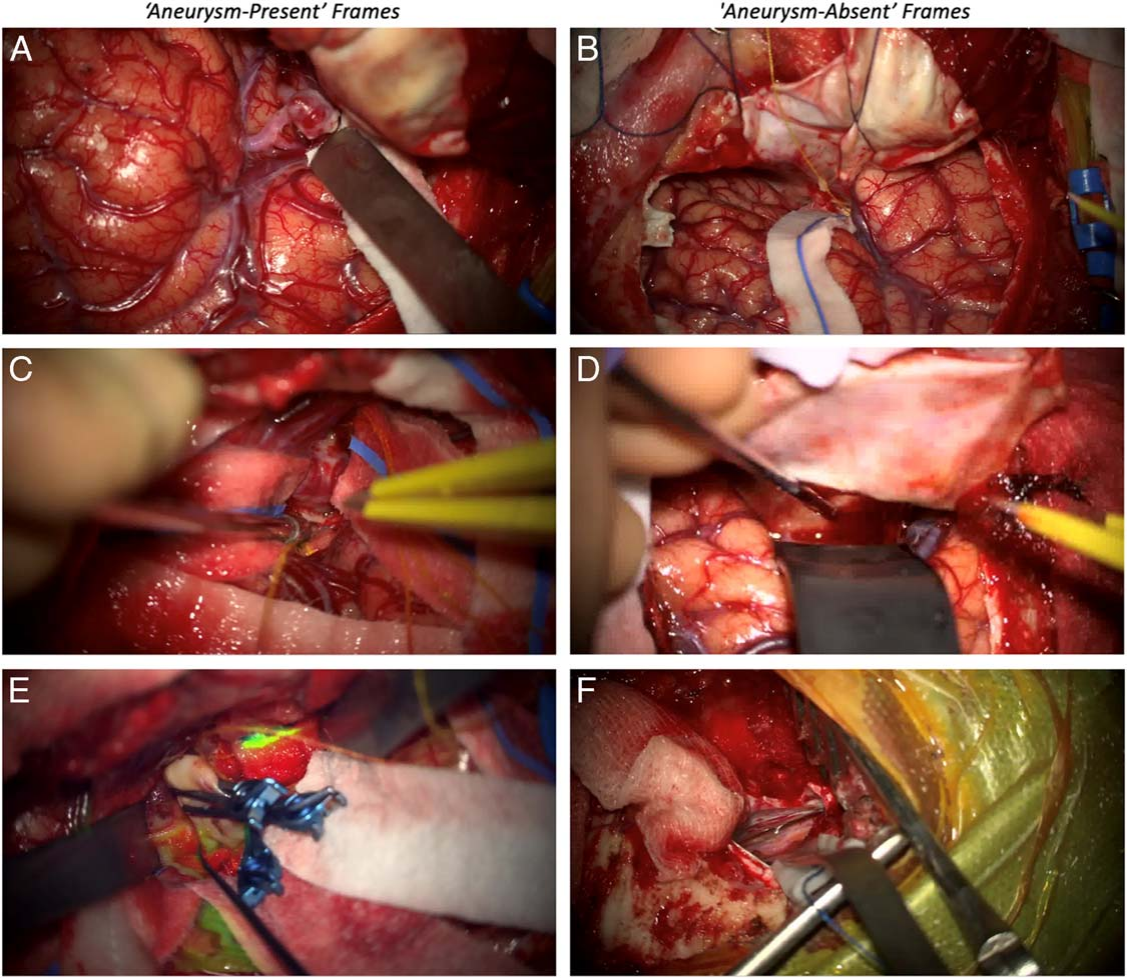
Examples of “aneurysm-present” frames (A, C, and E) and “aneurysm-absent” frames (B, D, and F).

Frames were extracted from the four operative videos (unseen to the MACSSwin-T platform) at a rate of 5 frames per second, resulting in 96,129 total frames (the Validation Data Set). A random number generator (Random Number Generator; randomwordgenerator.com/number) was used to select 50 frames. Ground truth was established through blinded review in duplicate by 2 vascular neurosurgeons where frames were classified as “aneurysm-present,” “aneurysm-absent,” or “exclude” (Fig. 1). Reasons for frame exclusion were:

- Microscope not pointing at the patient.
- Microscope moving.
- Indocyanine green angiography in process.
- Ambiguous image with partial view of the aneurysm making it inconclusive to assign either 'aneurysm-absent' or ‘aneurysm-present’ label.
- Instruments crossing the field of view.
- Rapid changing view within the scene.

To ensure accurate ground truth during frame reviews, at least one of the reviewers had been present during the operation from which the frames were derived, and reviewers had access to the operative videos. Frames with conflicting labels (6/50) were excluded. A final data set of 15 frames was selected from those with concordant reviews, to include 8 “aneurysm-absent” frames and 7 “aneurysm-present” frames (all frames can be found in Supplemental Digital Content 2, http://links.lww.com/SLA/F340). These 15 frames were used to create an online survey using SurveyMonkey (Momentive Inc.).

### Data Collection: Predictions From Health Care Professionals and MACSSwin-T Analysis

Human performance in aneurysm detection was assessed using an online survey, comprising an initial set of demographic questions, followed by the 15 still frames. Participants were asked to answer whether each frame did or did not contain a cerebral aneurysm in view, in a “yes” or “no” format. All participants reviewed the same 15 frames, in the same (randomized) order. The time to completion of the survey was recorded. Data collection was conducted over a 4-week period in September 2022. Participants were neurosurgical health care professionals defined as neurosurgeons and anesthetists of senior (attending/consultant) or junior (trainee/resident/fellow) grade, and OR nurses. Eligibility criteria for study participants included fulfilling one of these roles and working at a neurosurgical center. No criteria were applied regarding the neurosurgical subspecialty of practice. Participants were recruited from multiple centers internationally by local study collaborators. Collaborators were issued an information and instruction sheet including details of the research project, the host institution, and a QR code with a link to the survey.

Following the collection of responses from the neurosurgical team, the 15 frames were analyzed by the MACSSwin-T platform. The platform binarily classified each image as containing an aneurysm or not. Finally, a second independent round (round 2) of data collection was performed during a 4-week period in June 2023, using the same 15 frames, yet this time neurosurgical health care professionals were given the MACSSwin-T platform’s predictions for each frame, along with the gradient-weighted (GRAD) Cam class activation map for each frame. Human participants were informed that the platform has demonstrated an 81% accuracy in detecting cerebral aneurysms and was advised to use this to inform their decision-making.

### Exclusion Criteria

Incomplete survey responses (defined as <50% complete) were excluded, as were responses suspected to be falsified data.^24,25^ Quantitative steps can be employed to identify such data, and established methodologies such as those described by Hernandez and colleagues were employed to ensure the integrity of the data set (Supplemental Digital Content 1, http://links.lww.com/SLA/F340).^24,25^

### Data Analysis

Data were analyzed using Microsoft Excel (Microsoft Corporation), GraphPad (GraphPad Software, Inc.), and R (R Foundation for Statistical Computing). Standard definitions were used for accuracy, precision, recall, and F1 score.^4^ Comparative analysis was performed between humans only and humans with AI-assistance groups. Hierarchical mixed-effect regressions were employed for comparative analysis between groups with and without AI assistance, which enabled accounting for confounders and within-cluster correlations.^26^ The experimental group was set as the fixed-effect, and the random effects were frame nested within video, occupation, and trainee/expert status. For binary outcomes, mixed-effect logistic regressions were conducted. For a time, mixed-effected Gaussian regressions with log link functions were conducted. Two-sided CIs and *P* values were calculated. *P* values are reported to 2 significant figures up to values <0.001.^27^ The type-1 error rate was set at α <0.05, with Benjamini-Hochberge adjustments for multiple comparisons in any post hoc testing.^28^ Difficult and discrimination index for each frame was calculated and reported in Supplemental Digital Content 3 (http://links.lww.com/SLA/F340). For time-to-completion data, surveys that were recorded to have taken more than 60 minutes were removed, with the reasoning that these were likely interrupted sessions on surveys that are not likely to take more than an hour otherwise.

## RESULTS

### MACSSwin-T Performance

The MACSSwin-T platform binarily classified each frame as containing an aneurysm or not based on its predictive modeling. Results can be seen in the confusion matrix below (Table 1). The platform correctly predicted 87% (13/15) of all frames; 100% (8/8) of “aneurysm-absent” frames, and 71% (5/7) of “aneurysm-present” frames, giving an accuracy of 87%, precision of 100% recall of 71%, and an F1 score of 83%.

**TABLE 1:** Confusion Matrix for MACSSwin-T Model Performance on Fifteen Frames

	Ground Truth Positive	Ground Truth Negative
Prediction Positive	5	0
Prediction Negative	2	8

### Neurosurgical Team Performance: Round One (No Artificial Intelligence Assistance)

Round 1 (no AI assistance) included 230 responses after exclusions, comprising 3396 individual frame reviews. Complete responses (i.e. reviewed all 15 frames) were submitted by 208/230 participants; 22/230 submitted incomplete surveys, but with >50% frames completed thus eligible for inclusion. The survey was completed by 88 neurosurgeons (38 senior grade, 50 junior), 77 anesthetists (38 senior grade, 39 junior), and 65 OR nurses. Baseline characteristics of respondents can be found in Table 2.

**TABLE 2:** Baseline Characteristics of Respondents

	Round 1 (No AI Assistance)	Round 2 (AI-assisted)
Total respondents (n)	230	118
Sex (%)		
Male	54 (123/230)	45 (53/118)
Female	46 (106/230)	55 (65/118)
Prefer not to say	0.4 (1/230)	0 (0/118)
Non-binary	0 (0/230)	0 (0/118)
Age group (%)		
18–24	3 (7/230)	2 (2/118)
25–34	44 (102/230)	50 (59/118)
35–44	30 (68/230)	22 (26/118)
45–54	12 (28/230)	14 (16/118)
55–64	10 (22/230)	10 (12/118)
65–74	1 (3/230)	3 (3//118)
Occupation		
Neurosurgeon	38% (88/230)	31% (36/118)
Consultant/attending (N)	38	12
Trainee/resident/fellow (N)	50	24
Anesthetist	33% (77/230)	31% (37/118)
Consultant/attending (N)	38	18
Trainee/resident/fellow (N)	39	24
OR nurse	28% (65/230)	34% (40/118)

Analyzing responses from all health care professional respondents reveals an accuracy of 70% (2370/3396), specificity of 70% (2370/3396), sensitivity of 75% (1191/1587), positive predictive value of 65% (1191/1821), and negative predictive value of 75% (1179/1575).

Results by specialty are shown in Table 3. Neurosurgeons demonstrated an accuracy of 76% (993/1303), compared with 67% (753/1127) for anesthetists, and 65% (624/966) for OR nurse frame reviews. There were significant differences in accuracy between the groups on Analysis of Variance (*P* = 0.005), with post hoc pairwise testing with Bejamini-Hochberg adjustment for false comparisons showing a significant difference between neurosurgeons versus anesthetists and OR nurses, but no significant difference between anesthetists and OR nurses. Respondents were nonsignificantly better at identifying “aneurysm-present” frames than “aneurysm-absent” frames (neurosurgeons: 81% vs 72%; anesthetists: 69% vs 65%; OR nurses: 74% vs 57%; *P* = 0.09).

**TABLE 3:** Percentage and Number of Correct Frame Reviews Per Specialty (%, N)

	All Frames			“Aneurysm-absent” Frames			“Aneurysm-present” Frames		
	Round 1 (no AI assistance)	Round 2 (AI-assisted)	OR CI; *P*	Round One (no AI assistance)	Round 2 (AI-assisted)	OR CI; *P*	Round 1 (no AI assistance)	Round 2 (AI-assisted)	OR CI; *P*
Neurosurgeons	76 (993/1303)	88 (473/538)	2.66 (1.62–4.39); ^*^<0.001	72 (500/694)	87 (251/288)	3.28 (1.70–6.31); ^*^<0.001	81 (493/609)	89 (222/250)	2.22 (1.23–4.00); ^*^0.0082
Consultant/attending grade	77 (438/566)	92 (166/180)	4.24 (1.63–11.1); ^*^0.0031	73 (220/301)	91 (87/96)	5.12 (1.50–17.5); ^*^0.0092	82 (218/265)	94 (79/84)	4.26 (1.25–14.5); ^*^0.02
Trainee/resident/fellow grade	75 (555/737)	86 (307/358)	2.24 (1.26–3.98); ^*^0.0056	71 (280/393)	85 (164/192)	1.03 (1.27–6.20); ^*^0.010	80 (275/344)	86 (143/166)	1.74 (0.90–3.38); 0.1
Anesthetists	67 (753/1127)	77 (475/620)	1.75 (1.32–2.34); ^*^<0.001	65 (389/602)	75 (238/331)	1.84 (1.18–2.87); ^*^0.0073	69 (364/525)	79 (227/289)	1.86 (1.19–2.90); ^*^0.0067
Consultant/attending grade	69 (387/559)	75 (196/261)	1.43 (0.95–2.13); 0.08	69 (205/298)	78 (108/139)	1.72 (0.88–1.21); 0.11	70 (182/261)	72 (88/122)	1.22 (0.56–2.68); 0.62
Trainee/resident/fellow grade	64 (366/568)	78 (279/359)	2.08 (1.41–3.06); ^*^<0.001	61 (184/304)	73 (140/192)	1.96 (1.09–3.54); ^*^0.025	69 (182/264)	83 (139/167)	2.50 (1.48–4.22); ^*^<0.001
OR nurse	65 (624/966)	70 (422/600)	1.34 (0.98–1.84); 0.066	57 (290/513)	69 (220/320)	1.86 (1.17–2.96); ^*^0.0088	74 (334/453)	72 (202/280)	0.91 (0.58–1.42); 0.67

^*^ Statistical significance.

### Neurosurgical Team Performance: Round Two (Artificial Intelligence–assisted)

The data set for round 2 (AI-assisted) included 118 responses, comprising 1758 individual frame reviews. Complete responses (i.e. reviewed all 15 frames) were submitted by 114/118 participants, and 4/118 submitted incomplete surveys, but with >50% frames completed thus eligible for inclusion. Baseline characteristics for respondents can be found in Table 2. Analyzing responses from all respondents reveals an accuracy of 78% (1370/1758), which was statistically significantly greater than for round 1 [without AI assistance; 70% (2370/3396); odds ratio 1.77, CI: 1.44–2.17, *P* < 0.001]. Specificity was 77% (719/939), sensitivity was 80% (651/819), positive predictive value was 75% (652/872), and negative predictive value was 81% (719/887). Results by specialty are shown in Table 3. Accuracy in frame prediction increased with AI assistance for neurosurgeons (76% correct in round 1 vs 88% in round 2; odds ratio: 2.66, CI: 1.62–4.39, *P* < 0.001), anesthetists (67% vs 77%; odds ratio: 1.75, CI: 1.32–2.34; *P* < 0.001), and nonsignificantly with OR nurses (65% vs 70%; Odds Ratio: 1.34, CI: 0.98–1.84, *P* = 0.066). Difficulty and discrimination index for each frame is reported in Supplemental Digital Content 3 (http://links.lww.com/SLA/F340).

#### Time to Survey Completion

Median time to complete the survey was 5.8 minutes [Interquartile Range (IQR)]: 4.5–8.4) in round 1 (no AI assistance), and 5.5 minutes (IQR: 3.5–7.9) in round 2 (AI-assisted; *P* = 0.16). Neurosurgeons were nonsignificantly quicker to complete the survey with AI assistance [round 2 time 5.0 minutes (IQR: 3.7–6.5)] than without AI assistance [round 1 time 6.1 minutes (IQR: 4.5–8.8); *P* = 0.26]. This improvement was driven by attending neurosurgeons, whose time to completion near-significantly improved with AI assistance [round 1 time 6.2 minutes (IQR: 4.9–8.8) vs round 2 time 4.0 minutes (IQR: 3.3–5.6); *P* = 0.060]. No significant difference in timing was noted for anesthetists or OR nurses when comparing AI versus no AI assistance.

## DISCUSSION

### Principal Findings

This study presents the key findings from a comparative study comparing the accuracy of a deep-learning platform and neurosurgical health care professionals (with and without AI assistance) in the detection of cerebral aneurysms from microsurgical aneurysm clipping operations.

First, our data demonstrate that AI-assisted human performance is superior to both human performance and AI performance alone. In this study, AI assistance was provided by offering the MACSSwin-T model’s prediction along with frame activation maps. Improvements in accuracy was noted for all 3 occupations—neurosurgeons improved from 76% to 88%, anesthetists 67% to 77%, and OR nurses 65% to 70%. These results serve as a quantitative demonstration of the breakdown in shared mental models within the operating theater, and support the use of AI technology to enhance collaborative orientation among the neurosurgical team.^29^ Breakdown in shared understanding is particularly pertinent in neurosurgery, a high-stress, high-risk specialty in which communication breakdown influences patient outcomes and risk of litigation.^30^

Second, and perhaps most significant—our findings suggest that the greatest benefits were observed in the most experienced health care professionals, neurosurgical attendings, whose frame accuracy significantly improved, and time-to-completion near-significantly benefited. This finding purports the value of clinician-AI collaboration in improving surgical safety and contradicts assertions that the benefits of AI assistance are confined to junior clinicians.^31^ The reasons for this are intriguing. One possible explanation pertains to the adage “the more you know, the more you see”. In 1993, Ericsson et al^32^ published their seminal paper on the attainment of expert performance through deliberate practice; a key upshot of expert performance is the ability to interpret and understand more with a given data set or situation than non-experts. In the case of small, obscured, and challenging aneurysms, AI assistance may aid experts in confirming their suspicion that an aneurysm is present in the field of view.

**Fig. 2:**
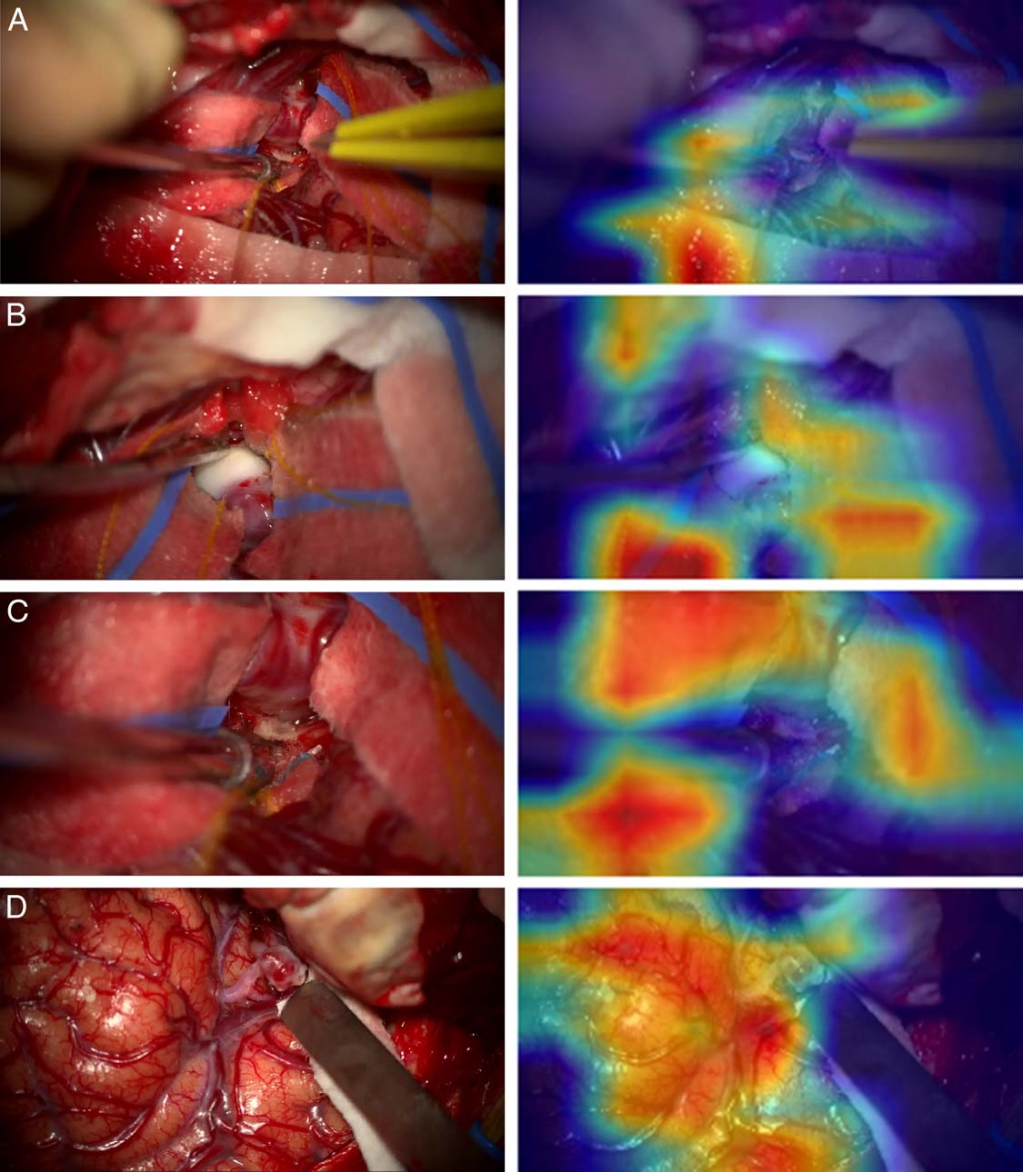
Challenging frames for MACSSwin-T and the neurosurgical team with associated MACSSwin-T Grad-CAM activation maps. A and B, “Aneurysm-present” frames that the MACSSwin-T platform incorrectly classified as “aneurysm-absent” frames. A, A complex operative scene with numerous instruments and vessels in the frame, as well as the aneurysm partially obscured by Cottonoids, which may account for the incorrect classification by MACSSwin-T. Human performance was also poor for this frame, with only 57% of the neurosurgical team correctly classifying the frame. B, The atherosclerosed aneurysm dome in clear view—this variation in appearance may account for the incorrect platform classification, as the activation map shows little focus on the white dome; in contrast, 85% of neurosurgeons correctly identified the aneurysm. C, An “aneurysm-present” frame correctly classified by the MACSSwin-T platform, yet was the frame on which human performance was worst, with 51% of respondents correctly identifying the frame as containing an aneurysm. D, The most commonly correctly classified frames by human assessors, with 84% of the neurosurgical team correctly labeling the “aneurysm-present” frame (the frame was also correctly classified by MACSSwin-T). Only 2 other frames had a greater correct percentage, both of which contained an aneurysm clip around the aneurysm.

Finally, we demonstrate that the MACSSwin-T platform outperformed humans (no AI assistance), and showed a high precision and recall in keeping with our previous work.^18^ Analysis of frame-level discrepancies between the MACSSwin-T platform and human performance reveals interesting findings. The platform was highly accurate in its identification of “aneurysm-absent” frames (100%), in part due to the imbalance in “aneurysm-absent” to “aneurysm-present” frames in the initial data set (80% vs 20%), as a result of aneurysm exposure making up only a small phase of the operation.^18^ Yet, with an inverse-proportional weighting of the 2 classes in the training loss function and adjustment of the model’s decision threshold, the platform was optimized to detect “aneurysm-present” frames without increasing erroneous “aneurysm-absent” detections (ie, false positives). This was apt in cases where the aneurysm was partially obscured, where humans found identification troublesome, but the platform was able to accurately locate the aneurysm (Fig. 2, frame C). Figure 2 shows challenging frames for MACSSwin-T and the neurosurgical team, along with activation maps demonstrating the focus point of the platform. “Aneurysm-absent” frames were particularly challenging for the neurosurgical team. Notably, correct identification of “aneurysm-absent” frames was increased with AI assistance, from 65% to 77%.

### Findings in the Context of the Literature

Current AI applications in the field of aneurysm detection primarily rely on radiomic-based methods, using imaging modalities such as Computed Tomography, Magnetic Resonance Imaging, or angiography.^33^ In this study, we present a novel approach to intraoperative aneurysm detection. While intraoperative computer vision has been successfully applied in several surgical contexts,^3,34,35^ its utilization in neurosurgery is emerging. Previous works by Pangal et al and Staartjes et al have demonstrated the feasibility of using deep-learning platforms for real-time anatomy segmentation in endoscopic endonasal surgery, in cadaveric and in vivo settings respectively.^36,37^ introduced the PAINet model for anatomic structure identification during pituitary surgery, achieving 66% accuracy in sella identification. Another significant contribution to neurosurgery comes from the study by Choi et al,^39^ who employed a “You Only Look At CoefficienTs” (YOLACT)-based architecture for anatomic detection in mastoidectomy. Our study adds to this growing body of research, expanding the potential applications of AI in neurosurgical procedures. Further, this study showcases the feasibility of an attention-based learning architecture (the shifted-window transformer model) in anatomic detection, distinguishing it from the prevailing use of convolutional neural networks.^34,39,40^ This substantiates an alternative, hierarchical strategy for developing novel AI architectures to tackle contemporary challenges in surgical contexts. Rapid, real-time anatomic recognition of intracranial aneurysms represents a leap in AI–health care capabilities, with numerous potential benefits in decision support, system efficiency, and education.

### Strengths and Limitations

This study has strengths in its international scope, adherence to established frameworks,^21,29,34,35^ and small-scale preclinical validation in a subset of neurosurgical attendings before wider distribution.^18^ Limitations include training on a limited data set from a single institution, reducing generalizability, and enhancing the risk of overfitting. Participants were asked to binarily classify frames as “aneurysm-absent” or “aneurysm-present,” whereas surgeons typically take a probabilistic approach to a live, three-dimensional situation. Some images were of low resolution, reflecting the challenge in the OR. Some participants commented that anatomy is more readily identifiable when benefiting from binocular disparity, enabling a three-dimensional view through the microscope. This is undoubtedly true, and we expect that neurosurgeons would score higher if able to view the operative scenes through a microscope and interact with the surgical environment. This does not, however, impact the ability of anesthetists or OR nurses who rely wholly on the microscope monitor. When selecting frames for the survey, frames with conflicting consultant labels were excluded, which may bias the selected frames towards easier instances. Data collection was conducted in the same centers for rounds 1 and 2, raising the potential for participants who had already completed the survey in round 1 to repeat it. To minimize this potential for bias, participants were not shown which frames they scored correctly/incorrectly after round 1, participants were asked if they had completed the survey before, and rounds 1 and 2 were conducted 9 months apart as a washout period. Due to anonymity requirements, we could not link individual scores between survey rounds, but we conducted a sensitivity analysis by accounting for whether participants had previously undertaken the survey as a random effect, which revealed no differences in the study’s results. Survey distribution in English may have introduced selection bias.

## CONCLUSIONS

This IDEAL stage 0 study compared a novel deep-learning computer vision platform (MACSSwin-T) with neurosurgical health care professionals in identifying cerebral aneurysms from microsurgical clipping operation images. Our data demonstrate that AI-assisted human performance is superior to human performance without AI assistance. Senior neurosurgeons benefited the most from AI-assisted aneurysm detection, with improved frame accuracy and time-to-completion. This research contradicts the prevailing narrative within the AI-health care paradigm, which asserts that the benefits of AI assistance are most notable in junior clinicians. Future research in this area should focus on model architecture iteration before first-in-human validation, in accordance with IDEAL stage 0 and 1 evaluation.

## Supplementary Material
