# Supplementary material for: Artificial Intelligence–Assisted Surgical Scene Recognition: A Comparative Study Among Health Care Professionals
Source: Ann Surg. 2024 Oct 30;283(5):760–8. doi: 10.1097/SLA.0000000000006577 (PMC13056406; doi:10.1097/SLA.0000000000006577)
Supplement: Supplementary file 1 [file sla-283-760-s001.docx]

**Supplemental Digital Content 1 – Individual Frames**

****Supplemental Figure 1:** Individual frames used within the study labelled with their ground truth: aneurysm absent or present**


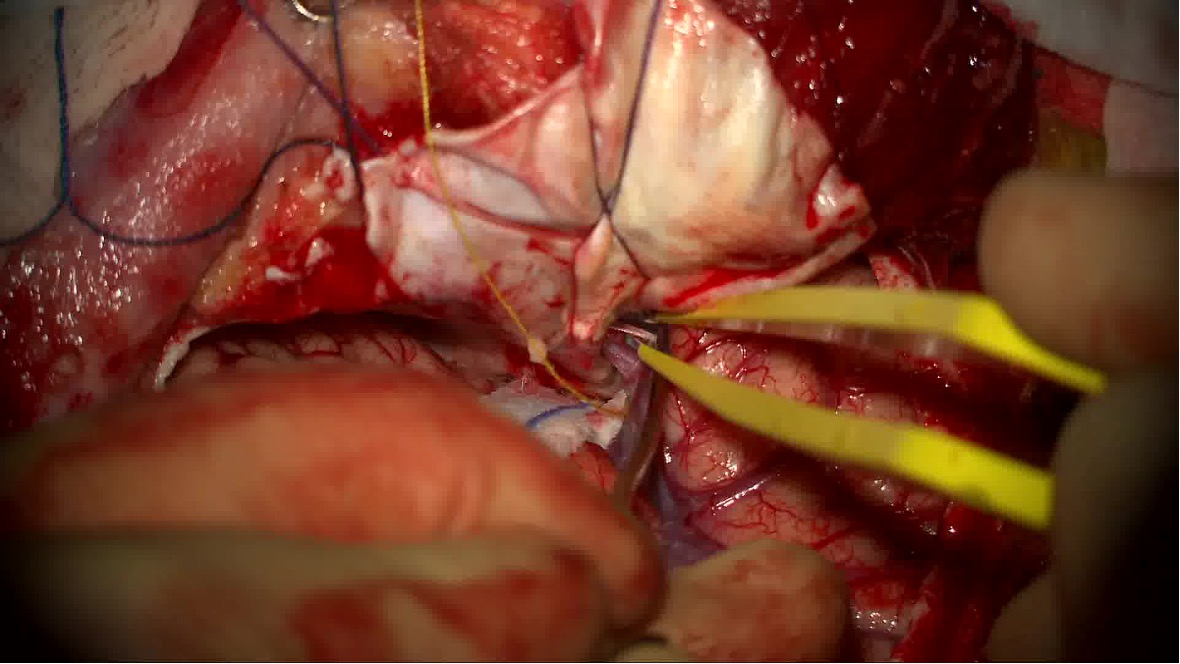


**Frame 1 (aneurysm absent)**


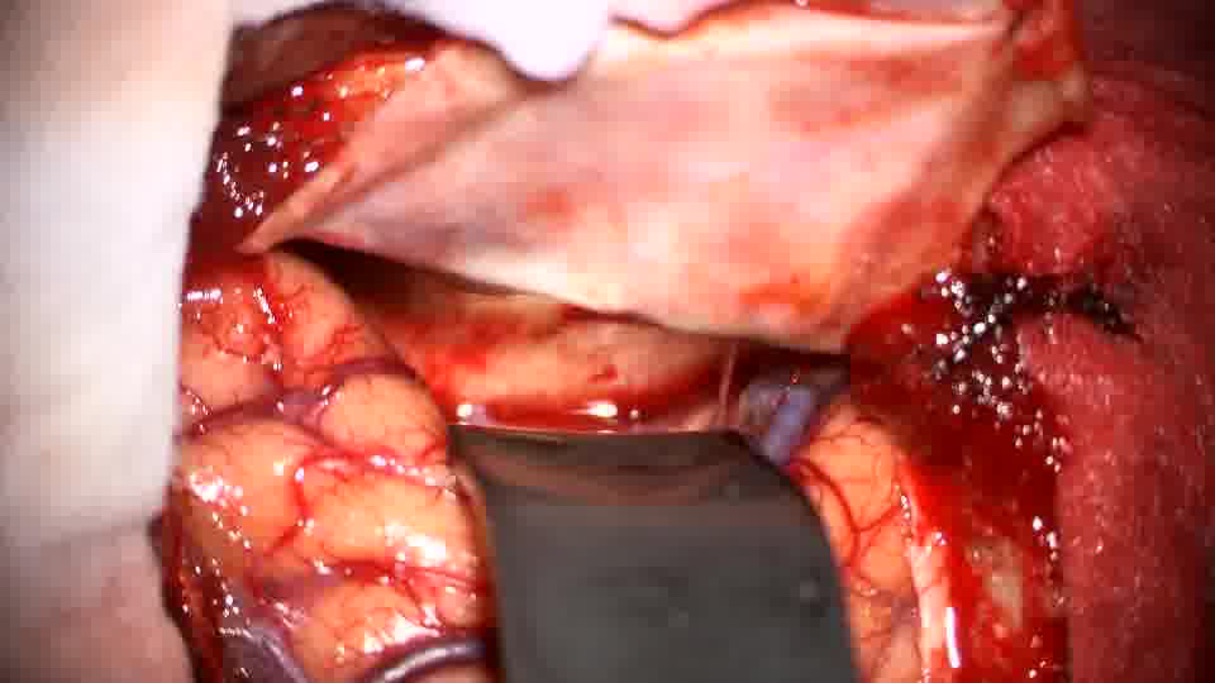


**Frame 2 (aneurysm absent)**


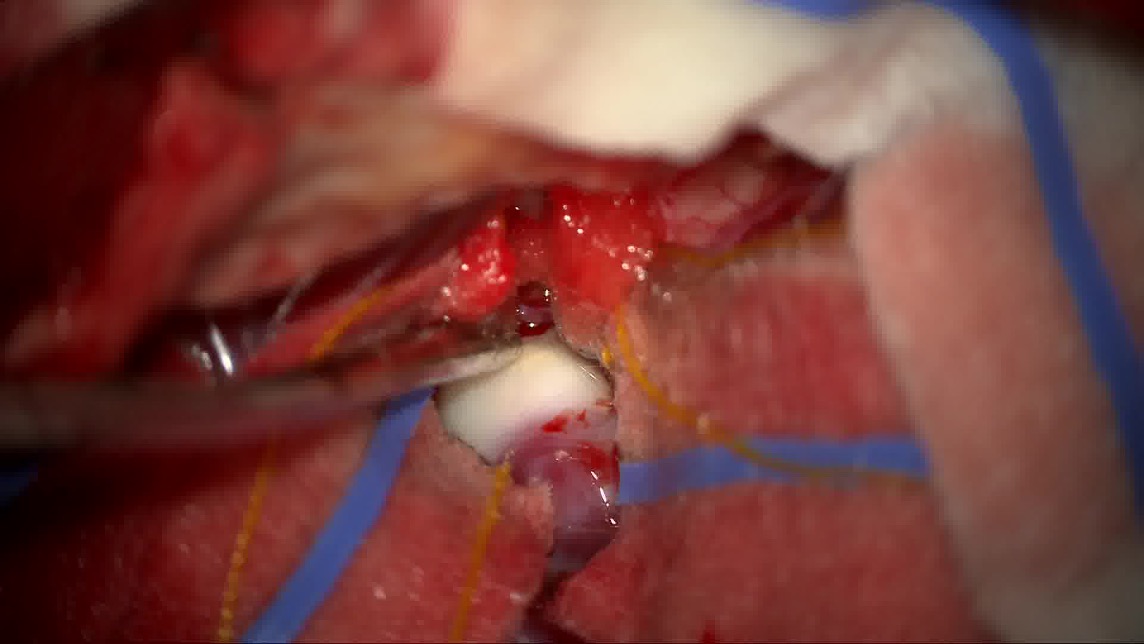


**Frame 3 (aneurysm present)**


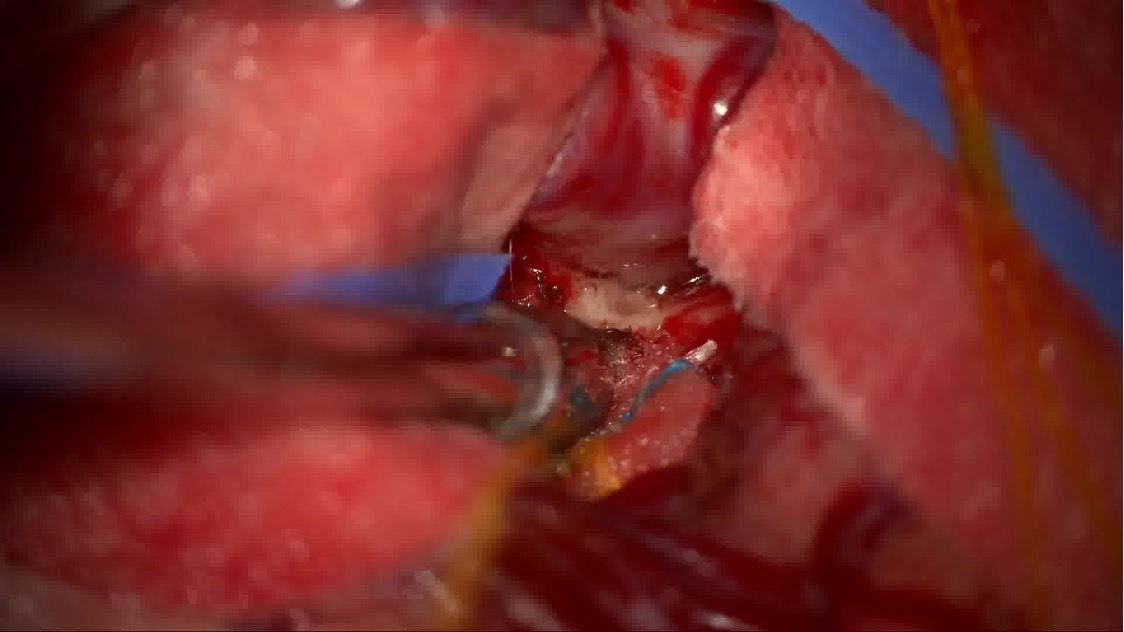

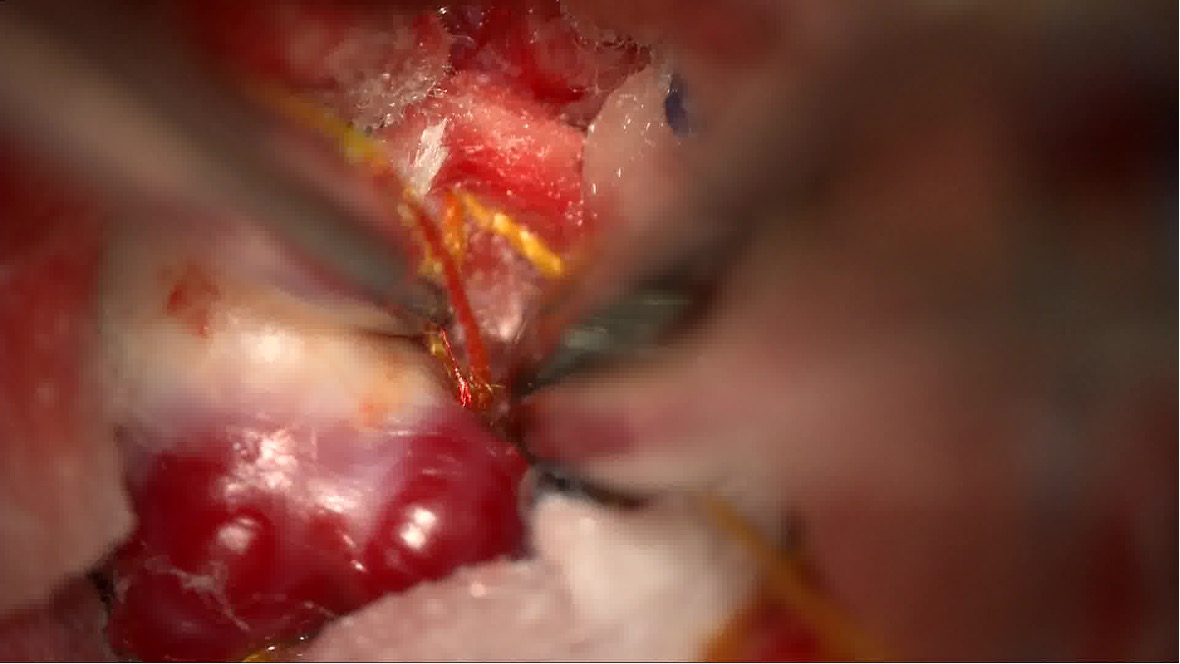

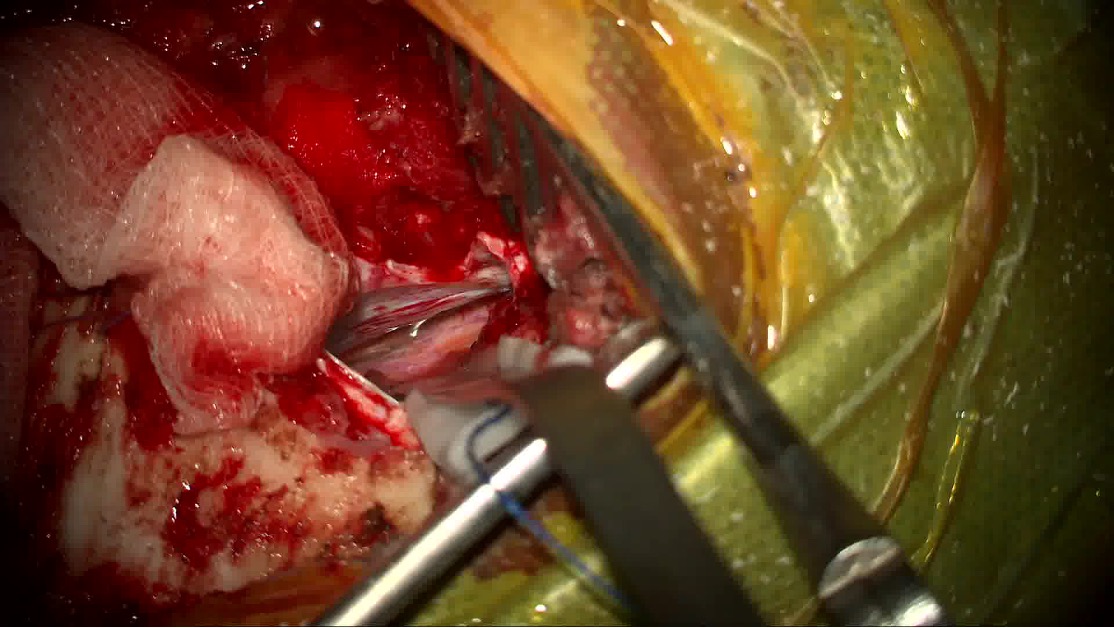


**Frame 4 (aneurysm absent)**

**Frame 5 (aneurysm present)**

**Frame 6 (aneurysm present)**


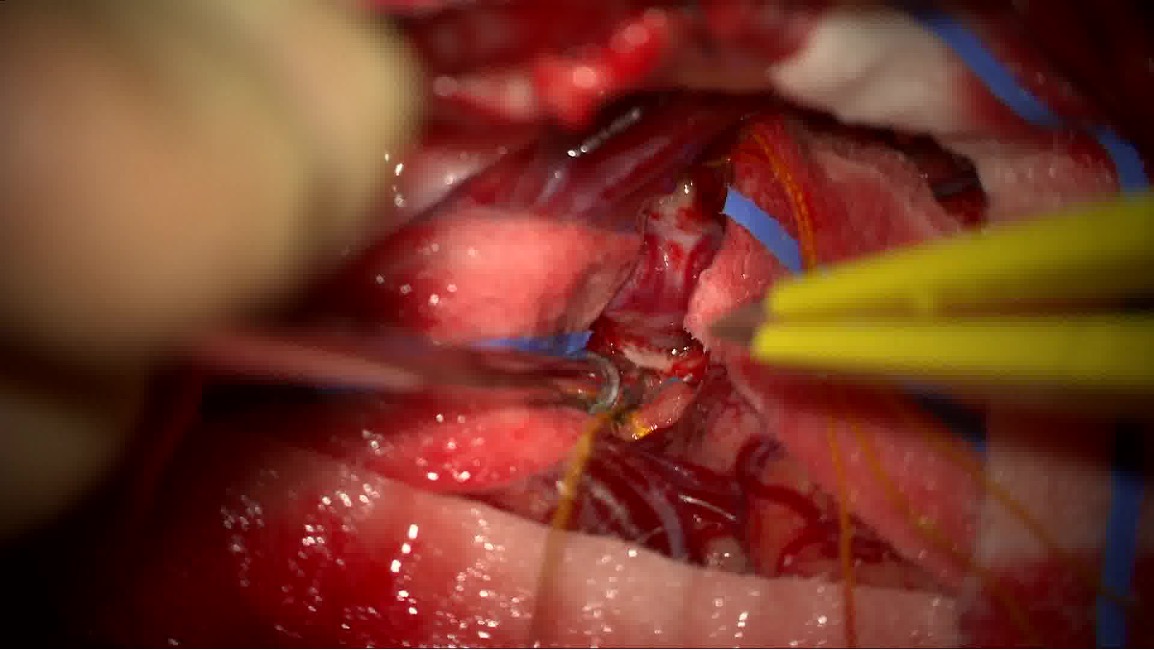

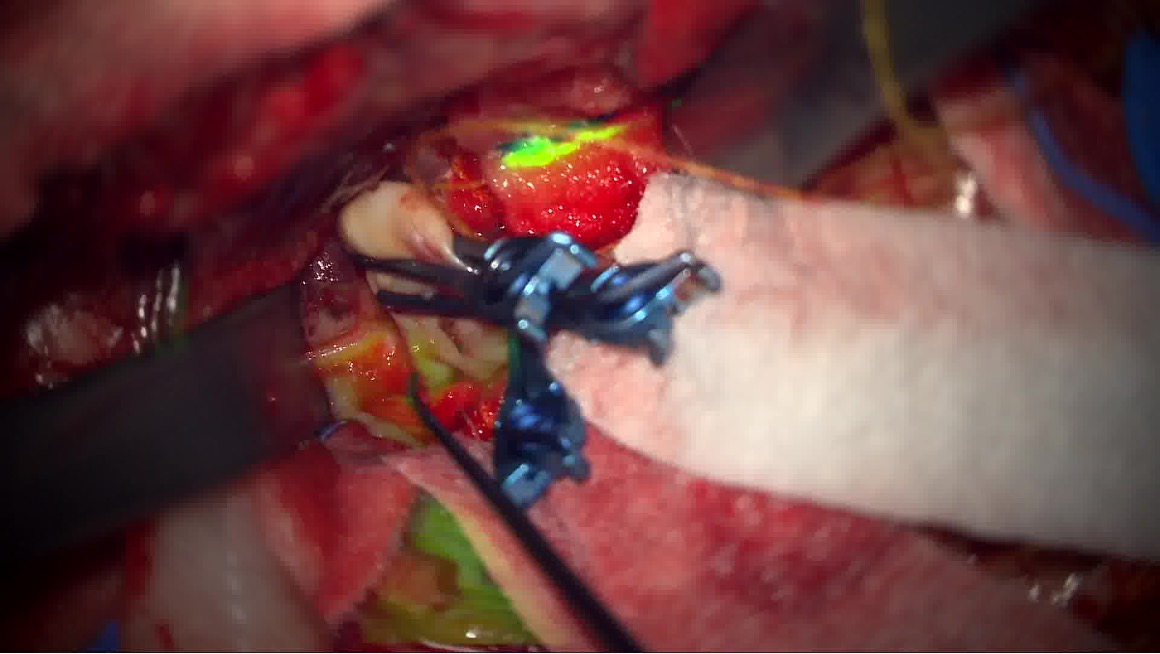

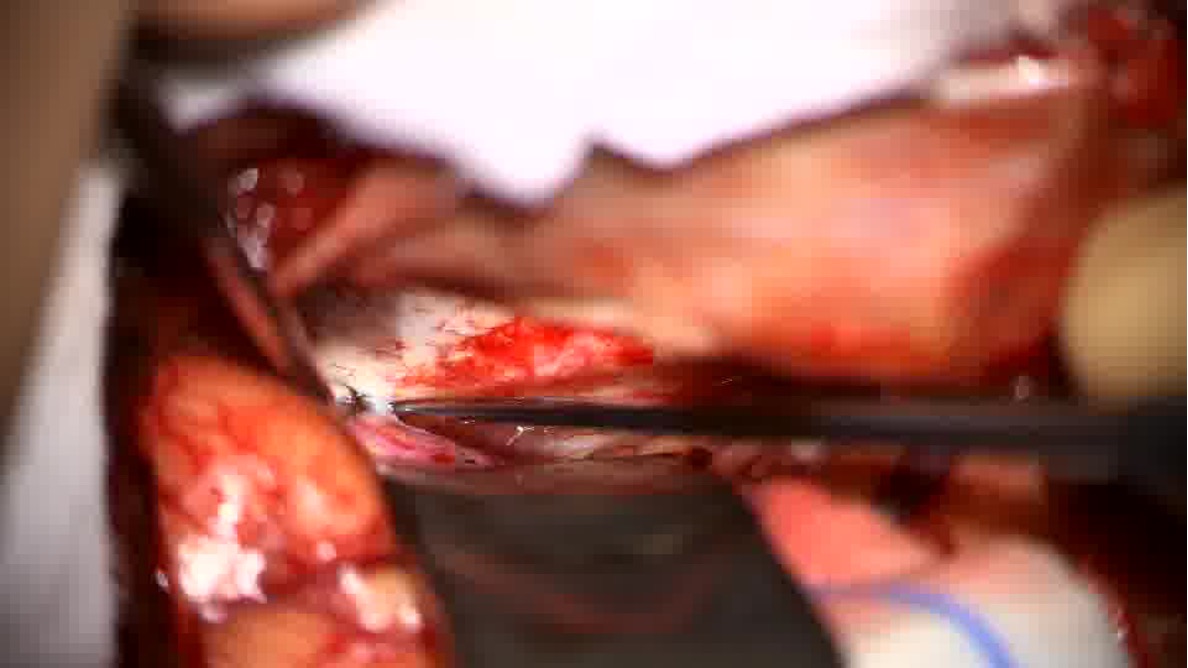


**Frame 7 (aneurysm absent)**

**Frame 8 (aneurysm present)**

**Frame 9 (aneurysm present)**


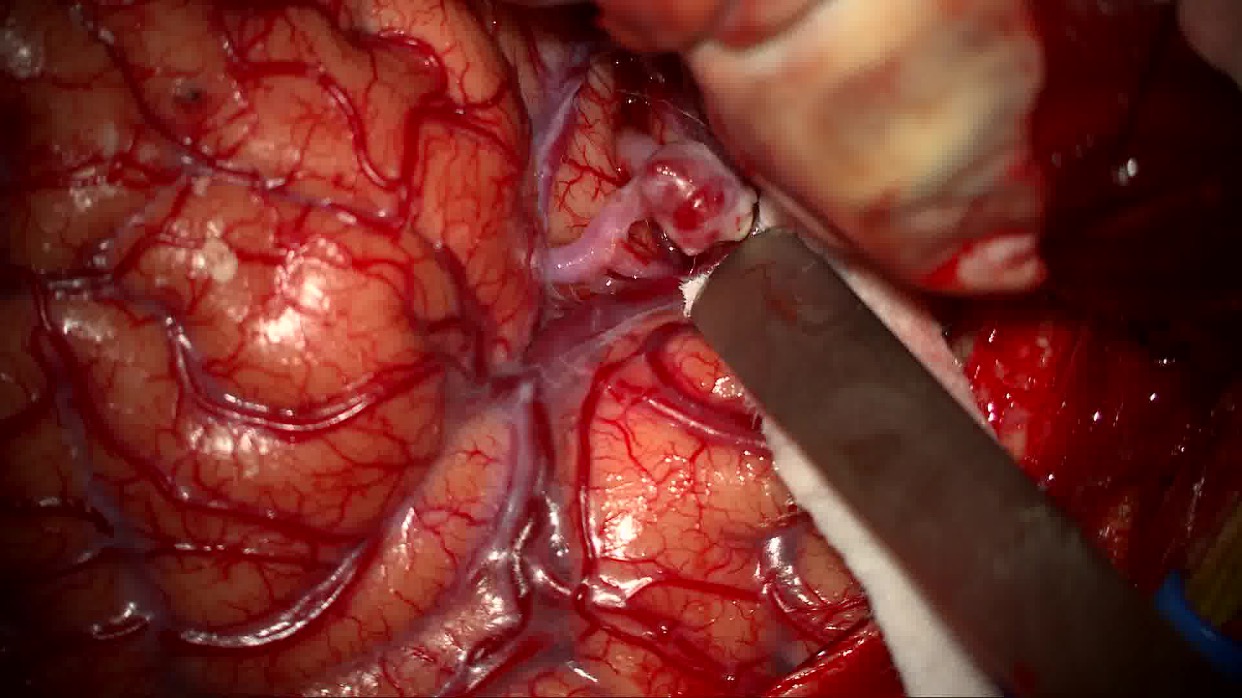

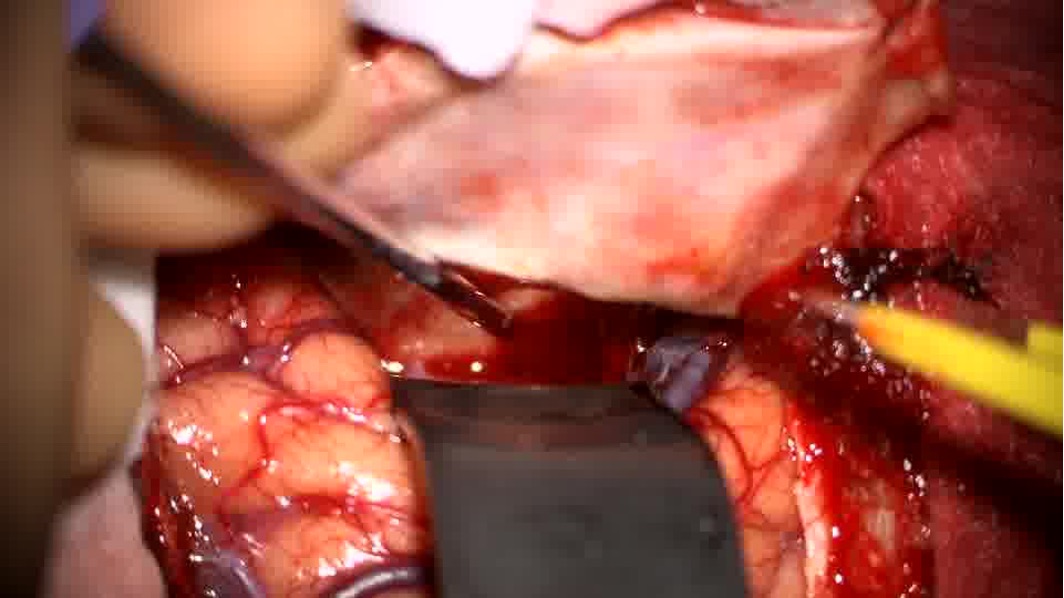

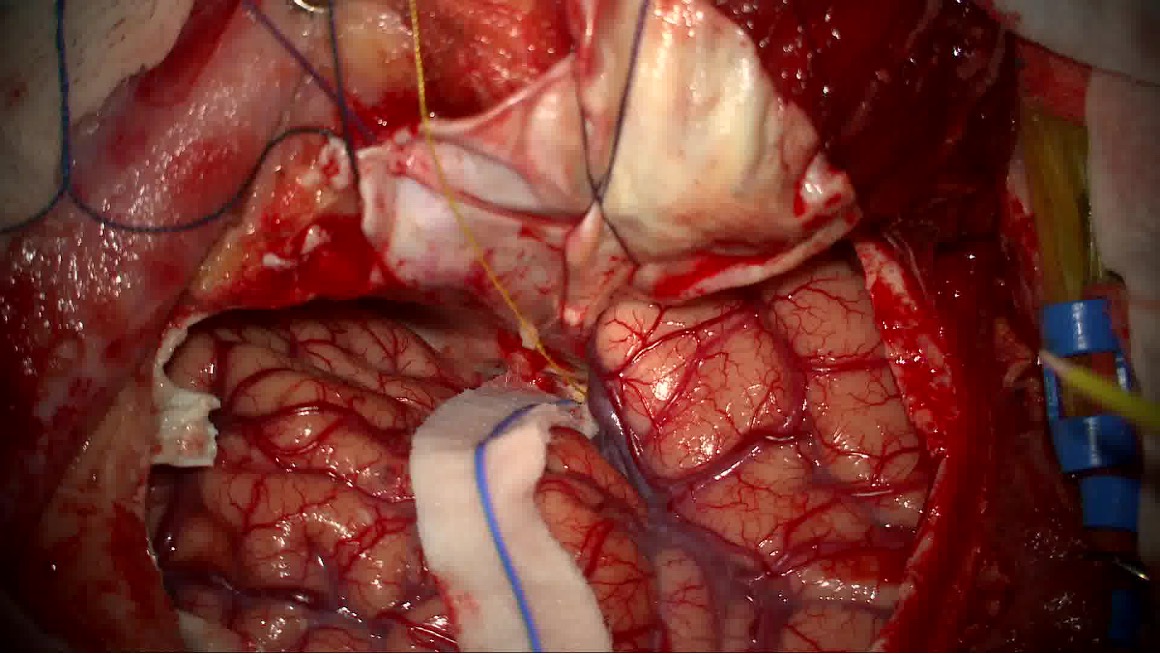


**Frame 10 (aneurysm absent)**

**Frame 11 (aneurysm absent)**

**Frame 12 (aneurysm present)**


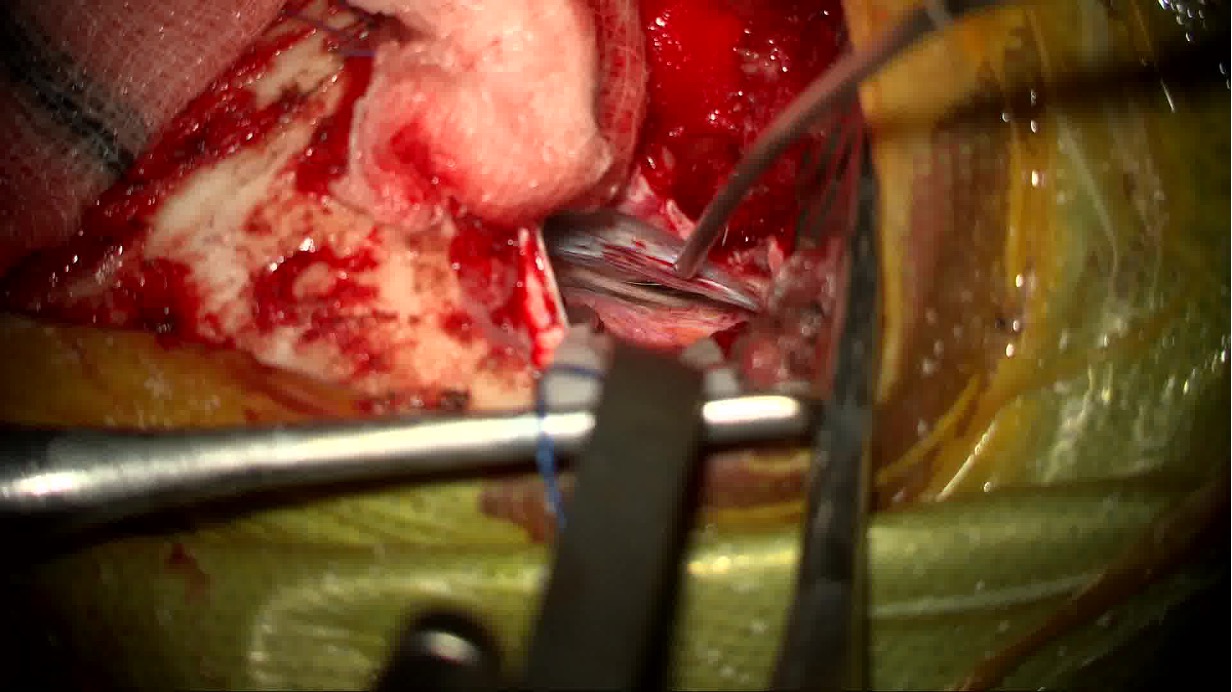

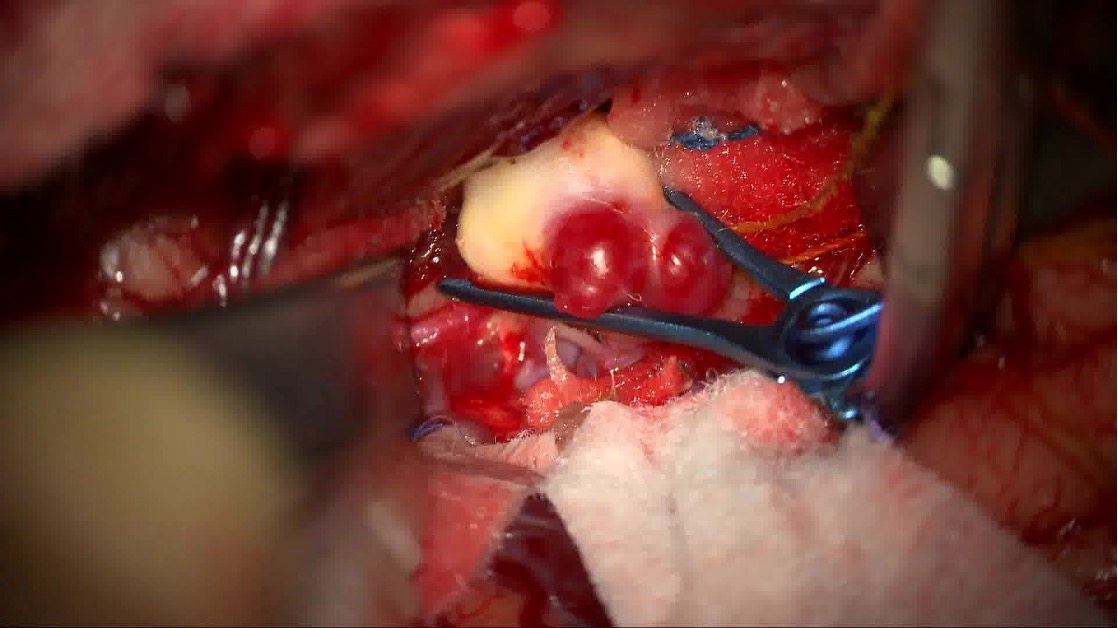

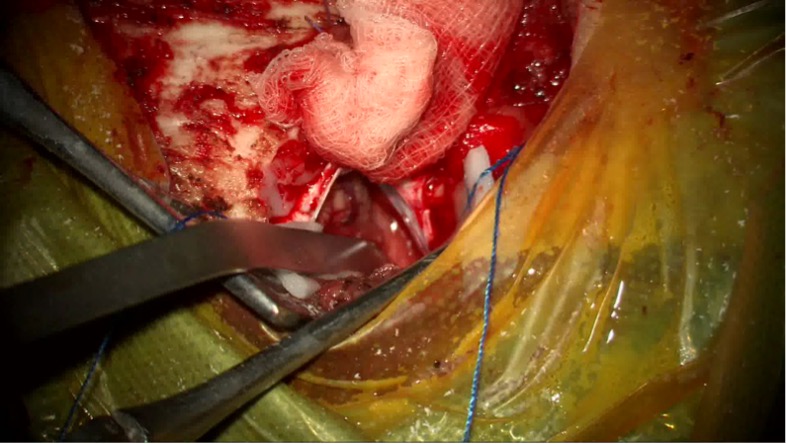


**Frame 13 (aneurysm absent)**

**Frame 14 (aneurysm present)**

**Frame 15 (aneurysm absent)**

**Supplemental Digital Content 2 - Exclusions and Detection of Falsified Data**

Incomplete survey responses (defined as <50% complete) were excluded, as were responses suspected to be falsified data. Falsification of data in research involving surveys is unfortunately a well-documented phenomenon.^19,20^ However, numerous quantitative steps can be employed to identify such data, in keeping existing validated methodologies.^19,20^ Detection of falsified data was based upon four quantitative metrics: (1) pattern of response submission, (2) response time for individual submissions, (3) Internet Protocol (IP) address homogeneity, and (4) response selection. If a collaborator’s responses were flagged as high risk they underwent manual review by the research team. Submissions deemed highly likely to be falsified were excluded from analysis.

Ninety-one collaborators signed up to participate in the study; 83 were contacted to take part in data collection (8 excluded due to duplication of centre); 29 collaborators submitted data; of these, four collaborators responses were flagged as high risk, prompting manual review. All four were excluded. Justification for exclusion is demonstrated below:

1. Pattern of response submission: all responses submitted on the same day or numerous responses submitted on the deadline day for response submission were deemed high risk. Of the 103 suspected falsified submissions 71% (73/103) were submitted on the final day of eligible data collection.
2. Response time for individual submissions: the survey takes approximately 5 minutes to complete. Median response time for the falsified data was 1.62 minutes (IQR 1.22 – 2.68), whilst median response time for legitimate data was 5.48 minutes (IQR 3.63 – 7.76) (P < 0.0001, Mann Whitney U). Time to survey completion for suspected falsified data versus remaining data can be found in Supplemental Figure 2.
3. IP Address Homogeneity: collaborators with a large proportion of responses from the same IP address were flagged as high risk. The median proportion of responses submitted from the same IP address for suspected falsified data was 56% (IQR 45 - 86) compared to 24% (IQR 12 - 51%) in legitimate data.
4. Response selection: responses out-of-keeping with known typical distributions for commonplace survey questions were flagged as high risk.

**
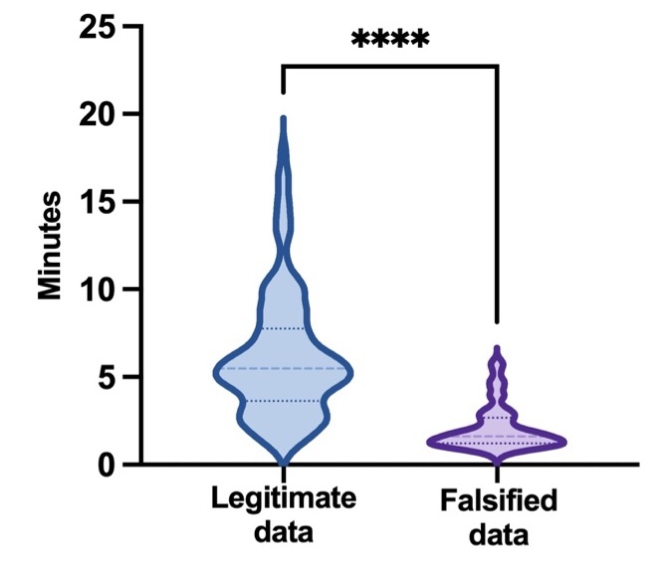
**

**Supplemental Figure 2: Time to Survey Completion for Suspected Falsified Data versus Remaining Data**

Once flagged as high risk, collaborator responses underwent manual review. Satisfaction of just one of the above criteria does not necessarily indicate that the data has been falsified, and therefore care was taken to investigate appropriately. In the case of the four excluded collaborators, three or more of the above criteria were met for each collaborator.

Falsification of survey data, termed ‘Curbstoning’, poses a significant problem to researchers conducting survey-based research. This publication supports the use of quantitative metrics in the detection of falsified data, enabling a higher-quality dataset to be achieved. Hernandez et al. describe numerous methodologies to prevent curbstoning.^1919^

**Supplemental Digital Content 3 – Difficulty and Discrimination Index of Each Frame**

Difficulty Index and Discrimination Index for Each Frame can be found in the Table below.

Difficulty index was defined as the proportion of correct answers for each individual frame.

Discrimination index was calculated by grouping individual responses into upper group performers (top 50% scores) and lower group performers (bottom 50% scores). The following formula was then used to calculate discrimination index: *DI*=(*U*−*L*)/*N , where DI = discrimination index; U = number of students in the upper group who answered correctly; L = number of students in the lower group who answered correctly; N = the total number of students.*

| **Table 4: Difficulty Index and Discrimination Index for Individual Frames** | | | | |
| --- | --- | --- | --- | --- |
|  | Round One (no AI) | | Round Two (AI assisted) | |
|  | Difficulty Index | Discrimination Index | Difficulty Index | Discrimination Index |
| Image 1  (aneurysm absent) | 0.70 | 0.34 | 0.78 | 0.13 |
| Image 2  (aneurysm absent) | 0.57 | 0.43 | 0.70 | 0.18 |
| Image 3  (aneurysm present) | 0.76 | 0.31 | 0.66 | 0.13 |
| Image 4  (aneurysm absent) | 0.66 | 0.33 | 0.68 | 0.21 |
| Image 5  (aneurysm present) | 0.79 | 0.25 | 0.81 | 0.13 |
| Image 6  (aneurysm present) | 0.51 | 0.05 | 0.65 | 0.18 |
| Image 7  (aneurysm absent) | 0.79 | 0.37 | 0.81 | 0.14 |
| Image 8  (aneurysm present) | 0.85 | 0.17 | 0.94 | 0.06 |
| Image 9  (aneurysm present) | 0.57 | 0.17 | 0.58 | 0.14 |
| Image 10  (aneurysm absent) | 0.54 | 0.26 | 0.73 | 0.17 |
| Image 11  (aneurysm absent) | 0.52 | 0.41 | 0.76 | 0.17 |
| Image 12  (aneurysm present) | 0.84 | 0.22 | 0.96 | 0.05 |
| Image 13  (aneurysm absent) | 0.65 | 0.33 | 0.81 | 0.11 |
| Image 14  (aneurysm present) | 0.93 | 0.17 | 0.97 | 0.03 |
| Image 15  (aneurysm absent) | 0.78 | 0.30 | 0.86 | 0.09 |
